# Immune-related ureteritis and cystitis induced by immune checkpoint inhibitors: Case report and literature review

**DOI:** 10.3389/fimmu.2022.1051577

**Published:** 2023-01-06

**Authors:** Jun Li, Ya-Fen Yu, Xiao-Wei Qi, Yuan Du, Chun-Qing Li

**Affiliations:** ^1^ Department of Nephrology, Affiliated Hospital of Jiangnan University, Wuxi, Jiangsu, China; ^2^ Wuxi School of Medicine, Jiangnan University, Wuxi, Jiangsu, China; ^3^ Department of Pathology, Affiliated Hospital of Jiangnan University, Wuxi, Jiangsu, China

**Keywords:** immune checkpoint inhibitors, immune-related adverse, glucocorticiods, case report, literature review

## Abstract

Immune checkpoint inhibitors (ICIs), including anti-cytotoxic T lymphocyte-associated protein 4 (anti-CTLA4) and anti-programmed death cell protein 1 (anti-PD-1), are increasingly prescribed in metastatic carcinoma therapy. ICI-related kidney injury is gradually recognized by clinicians. However, immune-related ureteritis and cystitis easily go undiagnosed. We report three cases of PD-1 monoclonal antibody (mAb)-related ureteritis and cystitis. We further carried out a review of the literature about ICI-related ureteritis and cystitis. The cases in our reports manifest urinary irritation, sterile pyuria, gross hematuria, hydronephrosis, dilation of the ureters, and acute kidney injury. Urinary irritation improved effectively; urinalysis and renal function returned to normal after glucocorticoid therapy. During ICI therapy, urinalysis and renal function and urinary imaging examination are recommended to be monitored regularly. It contributes to identify immune-related ureteritis/cystitis earlier to efficiently alleviate urinary symptoms and immunologic urinary tract injury through glucocorticoid therapy while avoiding the abuse of antibiotics.

## Introduction

Immune checkpoint inhibitors (ICIs), which disinhibit T-cell cytotoxicity against cancer *via* blocking cytotoxic T lymphocyte-associated protein 4 (CTLA-4), programmed death cell protein 1 (PD-1), or programmed death ligand-1 (PD-L1), are known to activate immunoactivity against malignancies (1, 2). ICIs include anti-CTLA-4, anti-PD-L1, and anti-PD-1 antibodies. As ICIs become more prevalent in cancer therapy, new and rare immune-related adverse events (irAEs) gradually attract the attention of medical oncologists. ICI-related acute kidney injury has been noticed in recent years, including acute interstitial nephritis, glomerulopathy (minimal change disease, focal segmental glomerulosclerosis, C3 nephropathy), and immune-related diseases (lupus nephritis, vasculitis, and thrombotic microangiopathy) (3–6).

However, irAEs involving the bladder and urinary tract are rarely reported and ignored by medical oncologists. The differential diagnosis of immune-related cystitis mainly includes bacterial cystitis, metastasis, radiation cystitis, and cystitis caused by other drugs. Obstructive nephropathy caused by renal calculus, carcinoma infiltration, and tuberculosis are excluded through CT scan. The diagnosis poses a challenge correspondingly. The patients usually received multiple courses of unnecessary antibiotics before getting an accurate diagnosis and treatment.

We reported three cases of immune-related ureteritis and cystitis, which were induced by ICIs [PD-1 monoclonal antibody (mAb)]. We further performed a review of the literature about ICI-related ureteritis and cystitis (**Table 1**). It contributes to identify immune-related ureteritis/cystitis earlier to efficiently alleviate urinary symptoms and immunologic urinary tract injury through glucocorticoid therapy while avoiding the abuse of antibiotics.

**Table 1:** Summary of 10 cases of immune checkpoint inhibitor-related cystitis.

Case No.	Age/Gender/Carcinoma	Immune checkpoint inhibitors	Pathology of urothelium	Dose of glucocorticoid	Treatment outcome	Courses of glucocorticoid
1 (7)	61-year-old man metastatic melanoma	four cycles of nivolumab (every 15 days)	Infiltration of CD3+ and CD20+ and PD1+ lymphocytes	Prednisolone 0.5 mg/kg/day	Improved after 7 days without relapse	Tapered after 1 month, weaned within 3 months
2 (8)	51-year-old man small cell lung cancer (SCLC)	five cycles of nivolumab(every 3 weeks)	Infiltration of CD3+ and CD8+ lymphocytes	Methylprednisolone 80 mg twice daily	Symptoms resolved after 3 days	Tapered over 6 weeks
3 (9)	67-year-old woman breast cancer (cT4bN1M1, Stage IV)	on day 97 after atezolizumab	Infiltration of CD8+ and intracellular antigen 1 (TIA-1) + lymphocytes in the urothelium eosinophilic infiltrations PD-L1 expression	Prednisolone 40 mg/day (1 mg/kg/day)	Improved 2 days after therapy	Tapered after 4 days
4 (10)	53-year-old man pulmonary adenocarcinoma (cT1cN3M1c, Stage IVB)	on the fifth day after the third course of sintilimab	Undone	Methylprednisolone (80 mg, 1 mg/kg/day)	Resolved after 17 days of corticosteroid treatment.	Eight weeks
5 (11)	50-year-old man lung squamous cell carcinoma (Stage IV)	seven cycles of nivolumab	Undone	Prednisolone 60 mg/day (1 mg/kg/day)	Alleviated after 3weeks	Unknown
6 (11)	60-year-old man lung squamous cell carcinoma (Stage IV)	12 courses of nivolumab administration	Undone	Not used	Resolved after nivolumab withdrawal	Unknown
7 (12)	47-year-old man pulmonary adenocarcinoma (Stage IV)	18 cycles of nivolumab	Infiltration of eosinophils and plasma cells	Not used	Alleviated after the bladder biopsy	Unknown
8 (13)	78-year-old woman lung adenocarcinoma (cT4bN3M1a)	six cycles of pembrolizumab	Infiltration of CD8, TIA-1-positive lymphocytes and positive PD-L1 expression in the urothelium	Prednisolone 25 mg/day	Alleviated after 19 days of treatment with steroid	Tapered within 2 months.
9 (14)	62-year-old man pulmonary squamous cell carcinoma (T4N0M1a, Stage IV)	On the 22nd day after administration of nivolumab	Mucosal epithelium completely sloughed off, interstitial edematous changes, slight lymphocytic infiltration	Steroid pulse therapy (methylprednisolone 500 mg × 3 days)	Resolved quickly	Maintenance dose 0.5 mg/kg/day, decreased gradually
10 (15)	48-year-old man intrahepatic cholangiocarcinoma (ICC)	Three cycles of nivolumab	Undone	Not used	Relieved after 3 months of drug withdrawal	Unknown
10 (16)	Case 10	Reoccurrence after three cycles of atezolizumab	Chronic inflammation of mucosal tissue, mucosal erosion, proliferation of granulation tissues and fibroblasts	Steroid hormones were given, which started at 2 mg/kg/day	Improved quickly	Unknown

TIA-1 (T cell intracellular antigen 1 or cytotoxic granule-associated RNA-binding protein); PD-L1, programmed death ligand-1.

## Case 1

A 49-year-old man was diagnosed as having esophageal carcinoma (PT3N3M0, Stage IV) in December 2020. Radical resection of midpiece esophageal carcinoma was performed, and chemotherapy with “paclitaxel (albumin-bound) + oxaliplatin” was given for four courses. In November 2021, chemotherapy combination with immunotherapy (tislelizumab 200 mg on day 1 + docetaxel 100 mg on day 2 + nedaplatin 100 mg on day 3) was given due to multiple lymph node metastasis in the mediastinum and posterior peritoneum. The date of the last chemotherapy was 28 June 2022. He did not have a medical history of hypertension, diabetes, or kidney disease.

He complained of gross hematuria, pollakiuria, painful micturition, and low back pain after six courses of tislelizumab (PD-1 mAb). Physical examination: T (Temperature) 36.4°C, P(Pulse) 85 bpm(beats per minute), R (Respiration) 16 bpm(breaths per minute), BP (Blood pressure) 125/75 mmHg. The abdomen was soft, no tenderness and rebound. His bilateral renal percussive pain was positive. There was no edema in the lower limbs.

**Figure 1:**
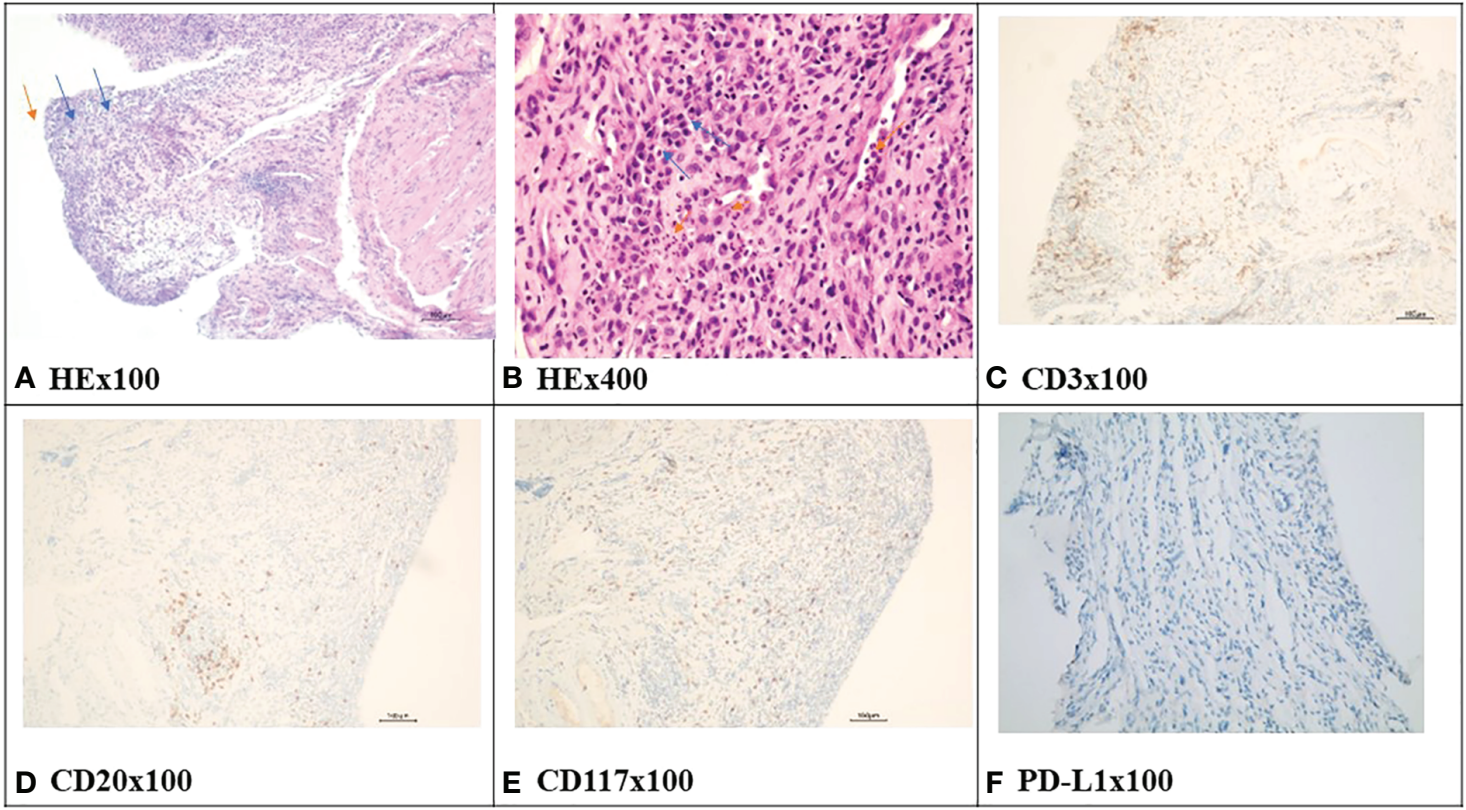
The HE and immunohistochemistry staining of bladder tissue in case 1. **(A)** showed effacement of the bladder urothelium (orange arrow), hyperplastic granulation tissue (blue arrow). **(B)** showed infiltration of monocytes, lymphocytes, plasmacytes (blue arrow), and neutrophils in the bladder tissue (orange arrow). **(C)** showed infiltration of CD3-positive lymphocytes in the bladder tissue (positive staining is brown). **(D)** showed positive infiltration of CD20-positive lymphocytes in the bladder tissue (positive staining is brown). **(E)** showed positive infiltration of CD117-positive mast cells in the bladder tissue (positive staining is brown). **(F)** showed that programmed death ligand-1(PD-L1) staining is negative in the bladder tissue. HE, hematoxylin-eosin.

**Figure 2:**
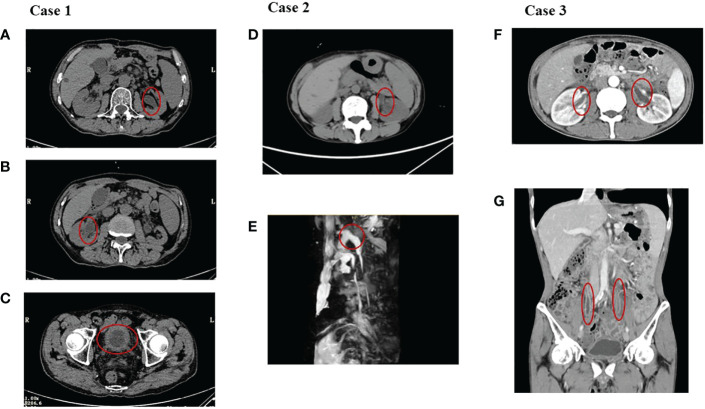
The images of the three cases. **(A–C)** showed hydronephrosis, dilation of the ureters, and a thickened bladder wall in case 1. **(D, E)** showed hydronephrosis and dilation of the ureter on the left in case 2. **(F, G)** showed dilation of the ureters and a thickened ureter wall in case 3.

**Figure 3:**
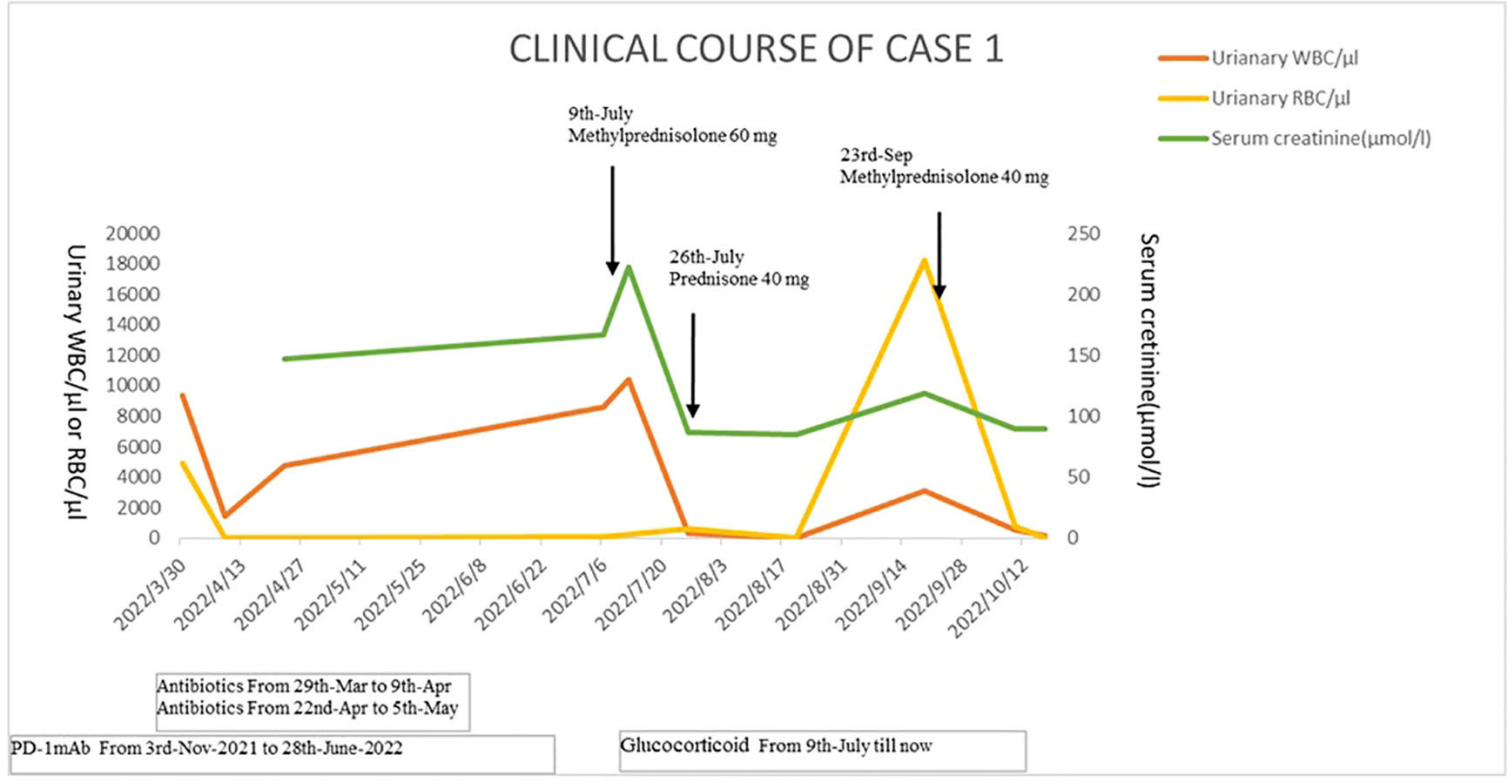
The timeline of the treatment course of case 1.

Urinalysis showed red blood cells (RBCs) of 4,932/µl, white blood cells (WBCs) of 9,375/µl, and proteinuria 3+. Biomarkers of renal tubular injury showed urinary N-acetyl-β-d glycosaminidase (NAG) 74.9 U/L (normal range 0.3–12 U/L), urinary neutrophil gelatinase-associated lipocalin (NGAL) 537.6 U/L (normal range 0.9–82 U/L), urinary albumin/creatinine 1,731.5 mg/g, urinary transferrin 1.74 mg/dl (normal range 0–0.2 mg/dl), and α1-microglobulin 0.95 mg/dl (normal range 0–1.25 mg/dl). Blood routine test showed that transient eosinophilia increased. Blood tests showed WBCs of 4,500/µl (neutrophils 35.6%, eosinophils 17%), lactate dehydrogenase 218 U/L, serum creatinine (sCr) 167 µmol/l (baseline sCr 81.7 µmol/l), erythrocyte sedimentation rate (ESR) 41 mm/h, and C-reactive protein <0.5 mg/L. The autoimmune antibody profile, immunoglobulin profile, and complement level were within normal range. Serum IgG4 was 0.497 g/l (normal range 0.03–2.01g/l). Fungal D-glucan test was negative. Serum procalcitonin was negative. Serum T-spot and urinary *Mycobacterium tuberculosis* were negative. Antibiotics were given, yet urinary symptoms were not relieved. His sCr level was significantly elevated to 211 µmol/l (baseline sCr 81.7 µmol/l). Blood tests showed that C-reactive protein was 41.7 mg/L; ESR was 120 mm/h. Repeated urine cultures were negative. Bilateral ureteral stenting was performed, and cystoscopy revealed diffused redness of the bladder mucosa, no sign of carcinoma infiltration. The pathologic change of the bladder tissue showed effacement of the bladder urothelium, hyperplastic granulation tissue, and infiltration of monocytes, lymphocytes, plasmacytes, and neutrophils in the bladder tissue. Immunohistochemistry staining of the bladder tissue showed positive staining of CD3, CD8, CD20, and CD117, yet negative staining of CD68, TIA-1, and PD-L1 in focal lesions (**Figures 1A–F**). The metagenomic next-generation sequencing (mNGS) of urine and ureter-bladder tissues was negative. Urinary ultrasonography and computed tomography showed mild hydronephrosis, dilated ureter, and thickened bladder wall (**Figures 2A–C**). Bladder residual urine was negative. The patient was eventually diagnosed as having immune-related ureteritis and cystitis on the 13th week after the occurrence of urinary symptoms. Methylprednisolone was administered at 60 mg (body weight 50 kg, equivalent to 1.5 mg/kg/day of prednisone) intravenously, and urinary irritation symptoms were relieved quickly after 3 days of treatment. After 2 weeks of methylprednisolone, sCr returned to the baseline level, and urinary WBC/RBC turned negative after 3 weeks of glucocorticoid therapy. Methylprednisolone was tapered gradually. Since the patient had a recurrence of urinary irritation symptoms, gross hematuria, and sterile pyuria on 16 September, methylprednisolone was increased to 40 mg intravenously, and the urinary irritation symptoms were alleviated quickly and urinary analysis improved. Bilateral ureteral stents were removed on 10 October 2022. The current dose of prednisone tablets is 25 mg. His urinary analysis showed that WBC was 145/μl, RBC was 5/μl, and sCr was 89.7μmol/l on 17 October 2022. The timeline of the treatment course was summarized in **Figure 3**.

## Case 2

A 62-year-old woman was diagnosed as having Stage IV gastric carcinoma in July 2020. From July 2020 to December 2020, oxaliplatin 150 mg plus tegafur-gimeracil-oteracil potassium capsules 40 mg (days 1–14) were administered for six courses, then tegafur-gimeracil-oteracil potassium capsules 40 mg days 1–14 were given alone in January 2021. Because of lymph node metastasis in the left clavicular and mediastinal area, she was given a regimen including sintilimab (PD-1 mAb) 200 mg, paclitaxel 200 mg (days 1–8), and tegafur-gimeracil-oteracil potassium capsules 40 mg (days 1–14) on 28 October 2022. Then, oxaliplatin 130 mg plus sintilimab (PD-1 mAb) 200 mg were given on 9 May 2022 and 1 June 2022. She did not have a medical history of hypertension, diabetes, or kidney disease.

She suffered from sudden-onset urinary irritation after three cycles of sintilimab (PD-1 mAb) treatment. Physical examination: T (Temperature) 36.3°C, P (Pulse) 70 bpm (beats per minute), R (Respiration) 16 bpm (breaths per minute), BP (Blood pressure) 110/60 mmHg. The abdomen was soft, no tenderness and rebound. Her renal percussive pain was negative. There was no edema in the lower limbs.

**Figure 4:**
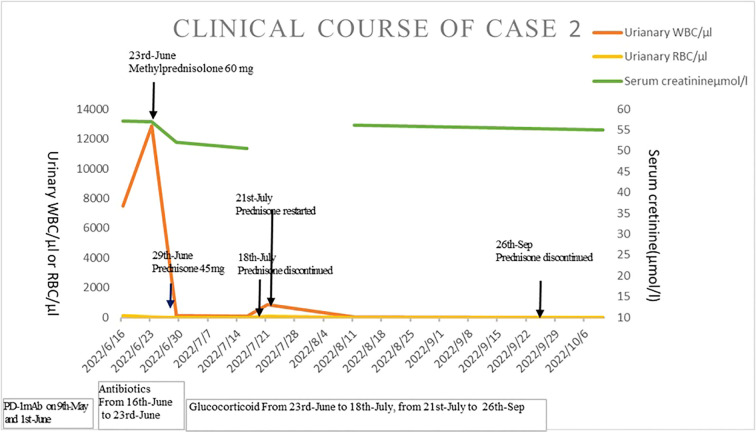
The timeline of the treatment course of case 2.

Urinalysis showed RBCs of 42/µl, WBCs of 17,916/µl, and proteinuria 3+. Blood tests showed WBCs of 2,300/µl (neutrophils 60.9%, eosinophils 4.3%) and C-reactive protein <0.5 mg/L. Her sCr was 56.2μmol/l (baseline sCr 56.4μmol/l). Serum T-spot and urinary *Mycobacterium tuberculosis* were negative. Repeated urine cultures were negative. Serum procalcitonin was negative. Biomarkers of renal tubular injury showed urinary NAG 29.2 U/L (0.3–12 U/L), urinary NGAL 247.6 U/L (normal range 0.9–82 U/L), urinary albumin/creatinine 466.9 mg/g, urinary transferrin 8.13 mg/dl (0–0.2 mg/dl), and α1-microglobulin 0.803 mg/dl (0–1.25 mg/dl). His 24-h urinary protein is 2.94 g. Urinary ultrasonography and CT showed mild hydronephrosis and dilation of the ureter on the left and a thickened bladder wall (**Figures 2D, E**). Bladder residual urine was negative. Antibiotics were given, and urinary symptoms were not relieved. The patient was eventually diagnosed as having immune-related ureteritis and cystitis. Methylprednisolone was given at 60 mg (body weight 44 kg, equivalent to 1.7 mg/kg/day of prednisone) intravenously after 18 days of urinary symptoms. After a week of methylprednisolone treatment, urinary symptoms were relieved and urinalysis was normal. The tapered prednisone was given at 45 mg and reduced by 5 mg per week. However, the patient discontinued prednisone by herself—assertion on 18 July 2022. And the urinary irritation symptoms and increased urinary WBC/RBC reoccurred on 21 July 2022. Prednisone was restarted at 30 mg/day and tapered gradually by 5 mg per week. Prednisone was discontinued on 26 September 2022. The last follow-up time was 10 October; urinary analysis remained negative and sCr was 55.5μmol/l. The timeline of the treatment course was summarized in **Figure 4**.

## Case 3

A 49-year-old man was diagnosed as having gastric carcinoma (PT2N1M0, Stage IV) in September 2021. Subtotal gastrectomy was performed in September 2021, then tegafur-gimeracil-oteracil potassium capsules were administered for six courses. Nivolumab 300 mg was given on 28 March 2022 and 18 April 2022. He did not have a medical history of hypertension, diabetes, or kidney disease.

Symptoms with hematuria, pollakiuria, painful micturition, and fever developed on the second day after the second course of nivolumab therapy. Physical examination: T (Temperature) 38°C, P(Pulse) 100 bpm (beats per minute), R (Respiration) 18 bpm (breaths per minute), BP (Blood pressure) 100/65 mmHg. The abdomen was soft, no tenderness and rebound. His bilateral renal percussive pain was positive. There was no edema in the lower limbs.

**Figure 5:**
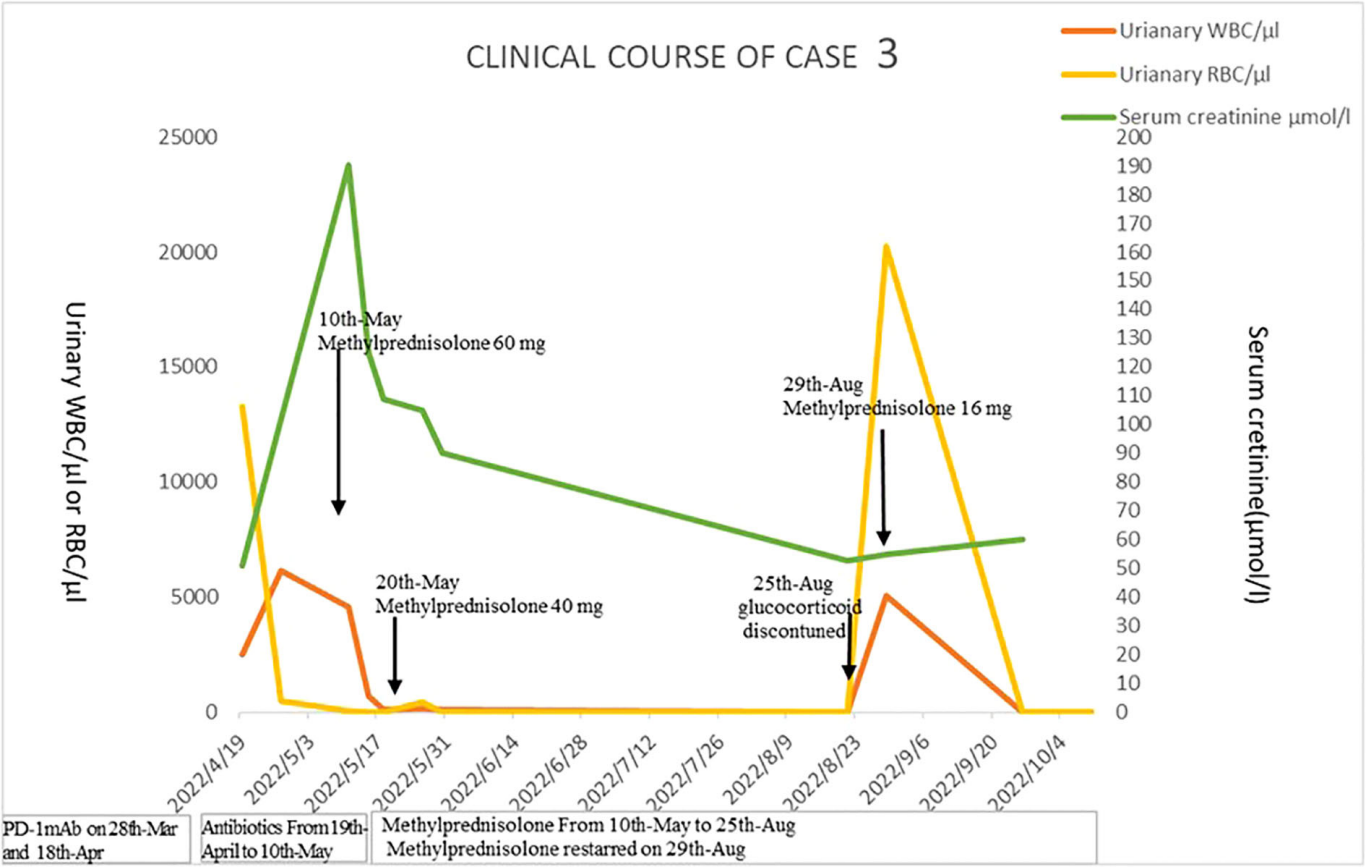
The timeline of the treatment course of case 3.

Urinalysis showed RBCs of 13,298/µl, WBCs of 2,506/µl, and proteinuria 3+. Urinary NGAL is 54.6 µ/l. Blood tests showed WBCs of 9,300/µl (neutrophils 72.8%, eosinophils 1.3%), C-reactive protein of 35.5 mg/L, lactate dehydrogenase of 164 U/L, sCr of 102 μmol/l, and ESR of 21 mm/h. Antibiotics were given. However, his sCr level significantly elevated to 190 µmol/l (baseline sCr 50.9 μmol/l). The autoimmune antibody profile, immunoglobulin profile, and complement level were within normal range. Fungal D-glucan test and limulus test were negative. Serum procalcitonin was 0.13 ng/ml. Serum T-spot and urinary *Mycobacterium tuberculosis* were negative. Blood culture was negative. Repeated urine cultures were negative. Urinary ultrasonography and CT showed mild hydronephrosis, dilated ureters, and thickened bladder wall (**Figures 2F, G**). Antibiotics were given, and urinary symptoms were not relieved. The patient was eventually diagnosed as having immune-related ureteritis and cystitis at the third week of urinary symptoms. Methylprednisolone was administered at 60 mg (body weight 44.5 kg, equivalent to 1.7 mg/kg/day of prednisone) intravenously, and body temperature returned to normal quickly and urinary irritation was relieved after 3 days of therapy. After 4 weeks of methylprednisolone, sCr returned to the baseline level and urinary WBC/RBC turned negative. The patient, who wished to initiate antitumor therapy earlier, complicated by intestinal fungal infections and steroid-induced diabetes during steroid treatment, reduced the dose of methylprednisolone quickly and discontinued it on 25 August 2022 by himself—assertion. Then, the patient had a recurrence of urinary irritation symptoms, gross hematuria, and sterile pyuria on 29 August. The methylprednisolone tablets were restarted at 16 mg, and the urinary irritation symptoms were alleviated quickly and urinary WBC/RBC returned to negative. The current dose of methylprednisolone tablets is 10 mg. Urinary WBC/RBC was negative from 26 September 2022 until now. His sCr was 60 μmol/l on 17 October 2022. The timeline of the treatment course was summarized in **Figure 5**.

## Discussion

We reported three cases of PD-1 mAb-related ureteritis and cystitis that were diagnosed as esophageal carcinoma (n = 1) or gastric carcinoma (n = 2). All three cases manifested significantly increased urinary WBC/RBC, and two cases had gross hematuria. CT scan of the three cases showed dilation of the ureters and hydronephrosis. Obstructive nephropathy caused by renal calculus, carcinoma infiltration, and tuberculosis were excluded through CT scan, and repeated urine cultures were negative. The detection of mNGS in urine and bladder tissue was also performed in case 1, and the mNGS reports were negative.

Two of the three cases were complicated by acute kidney injury after two or six cycles of PD-1 mAb infusion, whose renal function returned to baseline level gradually after steroid therapy. Although we did not perform renal biopsy, we speculate that their renal pathology was probably acute interstitial nephritis according to their good responses to glucocorticoid therapy (3–5).

During follow-up periods, the three cases all showed reoccurrence of sterile pyuria during glucocorticoid decrement. The rapid reduction of steroid might be the main cause of reoccurrence in both cases 2 and 3. However, severe pathologic lesions and more immune cell infiltration in bladder tissue might be due to four courses of PD-1 mAb infusion after the occurrence of immune-related ureteritis/cystitis in case 1. Moreover, ureteral stenting might aggravate the inflammation and edema of the urinary tract. Correspondingly, the patient’s urinary symptoms and urinary analysis improved quickly after the upregulation of glucocorticoid dose and removal of ureteral stents. Longer sessions of glucocorticoids are speculated to improve the immunologic urinary injury in case 1.

We further performed a review of the literature about ICI-related ureteritis and cystitis (7–15). Eight papers were retrieved to be reports about ICI-related cystitis and one paper about ICI-related ureteritis/cystitis (10).

Ten cases were reported to have 11 episodes of urinary irritation after ICI therapy. Of the 10 cases, acute kidney injury was complicated in one case, and immune-related cystitis reoccurred in one case after PD-1 mAb restarting (7–15).

The ICIs, which could induce immune-related cystitis, were mainly PD-1/PD-L1 blockers (n = 10), including nivolumab (PD-1 mAb, n = 7) (7, 8, 11, 12, 14, 15), pembrolizumab (PD-1 mAb, n = 1) (13), sintilimab (PD-1 mAb, n = 1) (10), and atezolizumab (PD-L1 mAb, n = 2) (9, 15). The three cases in our report were also diagnosed as PD-1 mAb-related ureteritis and cystitis. It seems that PD-1/PD-L1 pathway blockage might induce immune-related ureteritis and cystitis more commonly.

The expression of PD-L1 in bladder tissue was identified in patients with severe bladder inflammation (16). It is speculated that cytotoxic T-cell activation induced by PD-1/PD-L1 mAb might attack both carcinoma and normal urothelium with PD-L1 expression (9, 13). In case 1, there was CD3 (7, 8, 13), CD8 (8, 9, 13), and CD20 (7) positive expression in bladder tissue, as reported by previous case reports of immune-related cystitis. CD117-positive mast cells were identified in a focal lesion, which means that mast cells are also involved in the immune-related cystitis in case 1. We also performed immunohistochemistry staining of TIA-1, CD68, and PD-L1, and the results were negative. Consistent with the report by Zhu et al. (8), the negative expression of PD-L1 was detected in the urothelium of case 1. It might be due to the severe injury and shedding of the bladder urothelium.

Immune-related ureteritis and cystitis were also observed in autoimmune disease, including lupus, Sjögren’s syndrome, and vasculitis (17, 18), which suggested that the immune attack of PD-L1 is not the only immunopathogenesis of immune-related ureteritis/cystitis. The exact mechanism of immune-related ureteritis/cystitis needs further research. Biomarkers of ICI-related ureteritis/cystitis with high specificity are urgently needed.

IrAEs have been suggested to occur at any time but usually develop within the first few weeks to months after administration initiation (19). In previous case reports of ICI-related cystitis, the time from the initiation of therapy to the development of cystitis ranged from 2 to 12 months (the third to 18th cycles of ICIs) (7–15). In our report, the patients presented with urinary symptoms that ranged from 3 weeks to 15 weeks (the second to sixth cycles of PD-1 mAb therapy). Hence, it is vital to monitor the possibility of immune-related ureteritis/cystitis if there are any new-onset urinary symptoms and abnormal urinary examination during ICI therapy. In our report, the three cases did not manifest other irAEs including skin, endocrine, pulmonary, and gastrointestinal injury. It makes the early diagnosis of urinary irAEs a challenge. Therefore, urinalysis, renal function, and urinary imaging examination are recommended to be monitored regularly during ICI therapy. It contributes to identify immune-related ureteritis and cystitis earlier to avoid the abuse of antibiotics and occurrence of corresponding adverse effects.

In previous case reports of ICI-related ureteritis/cystitis, nine cases received glucocorticoid therapy, seven cases received corticosteroids at 1.7 mg/kg/day (8–11, 15), two cases received glucocorticoids at 0.5 mg/kg/day (7, 13), and one case received glucocorticoid pulse therapy (14). All cases acquired remission of urinary symptoms and normal urinalysis results. The glucocorticoid was tapered gradually within 2 months in most cases.

Since all three cases in our report presented with severe urinary irritation symptoms, and two cases were also complicated by acute kidney injury, the glucocorticoid therapy (methylprednisolone) was started at a dose of 60 mg/day^-1^ (1.5–2.0 mg/kg/day). Urethral pain and pollakiuria disappeared several days after initiating steroid therapy. Urinary WBC/RBC returned to negative within 1–4 weeks. Renal function returned to baseline level within about 1 month in cases 1 and 3. Rapid tapering of glucocorticoid dose might lead to the reoccurrence of urinary irAEs. The three cases in our report were recommended not to receive ICI treatment again.

According to the published literature and the experience acquired in our cases, the initial dose and treatment course of glucocorticoids can be evaluated according to the following points: ① the grade of urinary symptoms (19, 20); ② whether complicated with ICI-related acute interstitial nephritis or glomerulonephritis (20); ③ whether complicated by irAEs in other organs (20). The reoccurrence of immune-related ureteritis/cystitis might occur during rapid tapering of glucocorticoids.

## Conclusions

ICI-related ureteritis/cystitis is rarely reported, and the diagnosis poses a challenge. It is vital to monitor any new-onset urinary symptoms and abnormal urinalysis during ICI therapy to identify immune-related ureteritis/cystitis earlier. Urinalysis, renal function, and urinary imaging examination are recommended to be performed regularly during ICI therapy courses. It contributes to identify immune-related ureteritis/cystitis earlier to efficiently alleviate immunologic urinary tract injury through glucocorticoid therapy and avoid the abuse of antibiotics. It is necessary to follow patients closely during steroid decrement to make prompt treatment of reoccurrence.

## Data availability statement

The original contributions presented in the study are included in the article/supplementary material. Further inquiries can be directed to the corresponding author.

## Ethics statement

Written informed consent was obtained from the individual(s) for the publication of any potentially identifiable images or data included in this article.

## Author contributions

JL (First author and Corresponding author) contribute to do the design, clinical data collection, and write the manuscript. Y-FY (co-first author), contribute to revise the manuscript, equal contribution and share first authorship. X-WQ contributes to do immunohistochemistry staining of bladder tissue, read and evaluate the pathologic change of the case reported in our paper. YD and C-QL contribute to collect the clinical data of the patients. All authors contributed to the article and approved the submitted version.
